# Nephroprotective activity of natural products against chemical toxicants: The role of Nrf2/ARE signaling pathway

**DOI:** 10.1002/fsn3.2320

**Published:** 2021-05-01

**Authors:** Emad Molaei, Ali Molaei, Farshad Abedi, A. Wallace Hayes, Gholamreza Karimi

**Affiliations:** ^1^ Faculty of Pharmacy Mashhad University of Medical Sciences Mashhad Iran; ^2^ Faculty of Medicine Mashhad University of Medical Sciences Mashhad Iran; ^3^ University of South Florida College of Public Health Tampa FL USA; ^4^ Pharmaceutical Research Center Institute of Pharmaceutical Technology Mashhad University of Medical Sciences Mashhad Iran; ^5^ Department of Pharmacodynamics and Toxicology Faculty of Pharmacy Mashhad University of Medical Sciences Mashhad Iran

**Keywords:** herbal medicine, natural products, nephroprotective, nephrotoxicity, Nrf2, xenobiotics

## Abstract

Nephropathy can occur following exposure of the kidneys to oxidative stress. Oxidative stress is the result of reactive oxygen species (ROS) formation due to intracellular catabolism or exogenous toxicant exposure. Many natural products (NPs) with antioxidant properties have been used to demonstrate that oxidative damage‐induced nephrotoxicity can be ameliorated or at least reduced through stimulation of the nuclear factor erythroid 2‐related factor 2 (Nrf2) signaling pathway. Nrf2 is a basic leucine zipper (bZip) transcription factor that regulates gene expression of the antioxidant response elements (ARE). Nrf2 is involved in the cellular antioxidant‐detoxification machinery. Nrf2 activation is a major mechanism of nephroprotective activity for these NPs, which facilitates its entry into the nucleus, primarily by inhibiting Kelch like‐ECH‐associated protein 1 (Keap1). The purpose of this article was to review the peer‐reviewed literature of NPs that have shown mitigating effects on renal disorder by stimulating Nrf2 and thereby suggesting potential new therapeutic or prophylactic strategies against kidney‐damaging xenobiotics.

## Graphical abstract

The purpose of this article is to review the studies of NPs that have shown mitigating effects on renal disorder by stimulating Nrf2 and suggesting potential new therapeutic or prophylactic strategies against kidney pathogens.

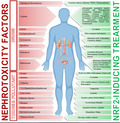

## 1 INTRODUCTION

The essential role of the kidneys is maintaining homeostasis. The kidneys do this by managing fluid levels, electrolyte balance, and other factors that keep the internal environment of the body consistent including excretion of toxic metabolites and drugs, regulation of osmolality, acid–base balance, hormone secretion, and blood pressure regulation (Finco, 1997). Renal dysfunction can occur following the use of certain medications or exposure to poisons (Barnett & Cummings, 2018). Some of the more important destructive renal effects are the result of induction of oxidative stress, followed by activation of nuclear factor kappa B (NF‐κB) and increased production of inflammatory cytokines such as nitric oxide (NO), tumor necrosis factor‐α (TNF‐α), interleukin‐1β (IL‐1β), IL‐6, and cyclooxygenase‐II (COX‐II). These changes can lead to an imbalance in homeostasis between these inflammatory factors and the antioxidant defense of the cell, which eventually leads to a pathophysiological disorder and apoptosis (Liu et al., 2017; Tak & Firestein, 2001).

It has been reported that many natural products (NPs) with antioxidant properties have beneficial effects in the amelioration of nephrotoxicity in different in vivo and in vitro models of kidney damage. Activation of nuclear factor erythroid 2 (NFE2)‐related factor 2 (Nrf2) has been suggested to plays a significant role in the protective effect of many of these antioxidant agents (Chen et al., 2016).

**FIGURE 1:**
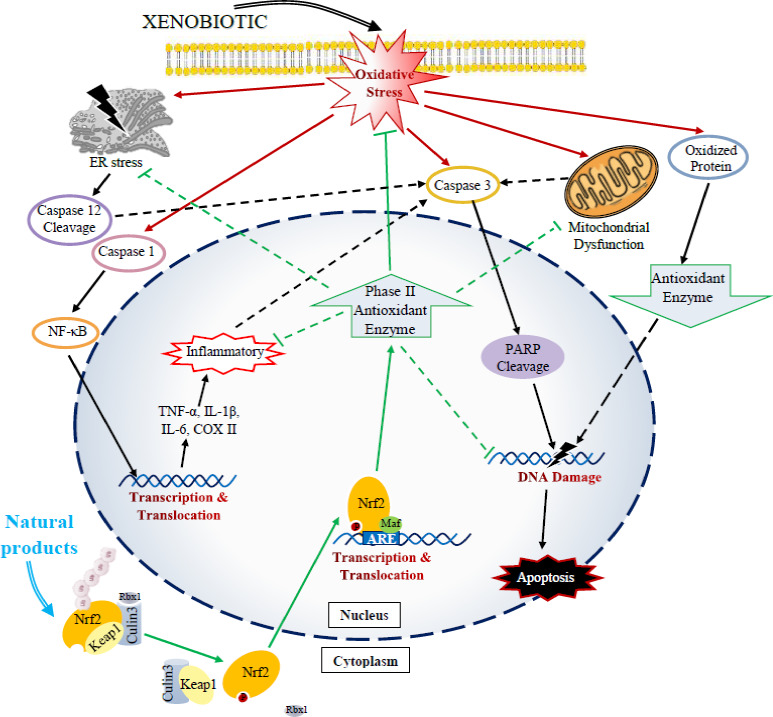
Mechanisms of nephroprotective activity of natural products by activating Nrf2 pathway against xenobiotics cytotoxicity

Nrf2, a basic‐region leucine zipper (bZIP) transcription factor encoded by the NFE2L2 gene, regulates the expression of sequences bearing the antioxidant response element (ARE) (Chan et al., 1995; Liu et al., 2009). Under normal physiological conditions, cytoplasmic Nrf2 is inactive in its complex with the Kelch like‐ECH‐associated protein 1 (Keap1) and Cullin 3 (Cul3). This binding targets Nrf2 for proteasomal degradation to maintain low intracellular levels of this molecular complex. Oxidative and electrophilic stress antagonizes the inhibitory effect of Keap1 on Nrf2 and disrupts the Keap1‐Cul3 ubiquitination system (Kansanen et al., 2013; Tonelli et al., 2018). Activated Nrf2 is translocated into the nucleus where a heterodimer is formed with small musculoaponeurotic fibrosarcoma proteins (Maf). The Nrf2 complex attaches to ARE which is located in the upstream promoter region of numerous genes, thereby encoding for the expression of antioxidant defense proteins (da Costa et al., 2019; Tebay et al., 2015). Factors produced in this signaling pathway include glutathione (GSH), glutathione peroxidase (GPx), and the thioredoxin antioxidant system and enzymes involved in phase‐I and phase‐II biotransformation of exogenous and endogenous products, nicotinamide adenine dinucleotide phosphate (NADPH) regeneration, and heme metabolism. The Nrf2/ARE signaling pathway plays a crucial role in cellular detoxification, antioxidation, and anti‐inflammatory effects (Iranshahy et al., 2018; Tavakkoli et al., 2019) (Figure 1).

We evaluated the peer‐reviewed literature for several natural products that have been reported to have a detoxifying and protective effect on the kidneys and classified them based on their potential nephroprotective activity.

## 2 METHODS

A comprehensive literature review was performed using the following keywords: “Nrf2” OR “nuclear factor erythroid 2 (NFE2)‐related factor 2” AND “nephrotoxicity” OR “kidney injury” OR “nephrotoxic” OR “nephroprotective” OR “nephroprotection” OR “Xenobiotics” AND “natural compounds” OR “natural product” OR “phytochemical” OR “phytomedicine” OR “phytomedicine” OR “herbal medicine” OR “medicinal herb” OR “plant extract” in the article title or list of keywords. The electronic databases used were as follows: Scopus, PubMed, Google Scholar, and Web of Science. The wild‐card asterisk (*) was utilized to enhance search accuracy. All bibliographies were reviewed to find relevant in vitro and in vivo studies published until February 2020. Only primary literature and not review articles were included. Duplicated, nonrelevant, and non‐English language articles were excluded. Following these search criteria, we found 119 articles in the online databases, among which 56 were excluded, while the remaining 63 articles were included in the review.

## 3 NRF2 AND DRUGS‐INDUCED NEPHROTOXICITY

### 3.1 Adriamycin

Adriamycin (Doxorubicin) is in the anthracycline and antitumor antibiotic family of medications used to treat breast cancer, bladder cancer, Kaposi's sarcoma, lymphoma, and acute lymphocytic leukemia. Adriamycin increases ROS, NO, and other free radicals and reduces detoxifying enzymes such as GSH, GPx, glutathione S‐transferase (GST), catalase (CAT), and superoxide dismutase (SOD), resulting in oxidative stress in the kidneys if not controlled. Adriamycin is an inducer of focal segmental glomerulosclerosis (FSGS) and other renal diseases (Tacar et al., 2013).

*Camelia Sinensis* (white tea) contains several polyphenolic compounds with antioxidant properties (Espinosa et al., 2014). Consumption of white tea has been reported to have beneficial physiological effects in the prevention and treatment of gastric cancer, cardiovascular disorders, and nervous system injury (Serafini et al., 2002). Long‐term administration of an aqueous white tea extract to mice protected the kidneys against oxidative damage of proteins and lipids caused by doxorubicin via activating the Nrf2/ARE signaling pathway. Interaction of Nrf2 with ARE after its upregulation accelerated the expression of detoxifying genes such as NAD(P)H quinone oxidoreductase 1 (NQO‐1), GST‐α_1_, and heme oxygenase 1 (HO‐1) with a significant positive effect on the activity of the antioxidant enzymes CAT, SOD, and glutathione reductase (GR) (Espinosa et al., 2016).

2,3,5,4′‐Tetrahydroxystilbene‐2‐O‐β‐D‐glucoside (THSG) is an active compound extracted from *Polygonum multiflorum Thunb* that is structurally similar to resveratrol. THSG belongs to the hydroxystilbene family of compounds. This NP is effective in improving the complications of aging (Ling & Xu, 2016), reducing disorders such as certain brain diseases, atherosclerosis (Zhang et al., 2008), diabetic nephropathy (Li, Cai, et al., 2010; Li, Huang, et al., 2010), and cardiac toxicity induced by doxorubicin (Zhang et al., 2009). An in vivo experiment showed that activation of Nrf2 by THSG protected the kidneys against the untoward effects of doxorubicin and reduced the expression of renal fibrotic genes by increasing NQO‐1, NAD(P)H_,_ and HO‐1 production. Another effect of boosting these enzymes is to suppress the oxidation of cell thiols and their damage (Lin, Bayarsengee, et al., 2018).

Thymoquinone, a phytochemical compound, is the most potent and pharmacologically bioactive constituent of *Nigella sativa* and/or black seed essential oil that has a wide spectrum of activities and therapeutic potentials such as antioxidant (Jrah Harzallah et al., 2012), anti‐inflammatory, anticancer (Woo et al., 2012), and antibacterial effects (Kouidhi et al., 2011). Histological analysis of renal tissue has shown that thymoquinone reduced NADPH oxidase 4 (NOX4)‐Nrf2 redox imbalance and doxorubicin‐induced nephrotoxicity in rats. Adjusting this imbalance included a reduction in the production of TNF‐α, IL‐6, and other inflammatory factors, an increase in certain cellular proteins such as glutathione‐S‐transferases (GST) and SOD, ultimately resulting in enhanced kidney resistance to the drug‐induced damage (Elsherbiny & El‐Sherbiny, 2014).

### 3.2 Cisplatin

Cisplatin is an antineoplastic medication used to treat several types of cancers. However, this drug has significant toxic side effects on several vital organs, especially the kidneys, which limit its therapeutic usefulness (Oun et al., 2018). The evidence strongly supports the hypothesis that oxidative stress and inflammation play an important role in the toxicity of cisplatin (Chirino & Pedraza‐Chaverri, 2009; Ojha et al., 2016). It has been suggested that antioxidants might reduce such side effects (Gómez‐Sierra et al., 2018).

*Andrographis paniculata* is an annual herbaceous plant used to treat liver disorders, cardiovascular damage, and infectious diseases among other clinical applications. The plant contains a wide range of chemical constituents, including lactones, flavonoids, and diterpenoids (Akbar, 2011). An ethanolic leaf extract was reported to protect the kidney against toxicity caused by cisplatin via the Nrf2/kidney injury molecule 1 (KIM‐1) cascade mechanism. More precisely, this extract downregulated the expression of the KIM‐1 protein, thereby inhibiting the activity of the phosphatidylinositol 3 (PI3) secondary messenger. In addition, the leaf extract of this plant separated Keap1 from inactivated Nrf2, and stimulation of this pathway increased the production of phase‐II detoxifying proteins. These changes eventually lead to reduced apoptosis and necrosis in kidney tissue (Adeoye et al., 2018).

Curcumin, another heptanoid NP, is a derivative of one of the polyphenolic compounds found in *Curcuma longs linn*. This substance has been reported to have beneficial therapeutic effects on several diseases such as metabolic syndrome, diabetic nephropathy, and neurological and liver disorders (Hatcher et al., 2008; Iranshahy et al., 2018; Trujillo et al., 2013). A curcumin and thymoquinone combination has been shown to be effective in alleviating renal side effects of cisplatin by regulating cellular protective proteins such as SOD, CAT, GPx, and HO‐1. The combination also suppressed the secretion of inflammatory factors such as IL‐6, TNF‐ α, NF‐κB, KIM‐1, and multidrug resistance protein (MRP)‐1. The study found that the combination of thymoquinone and curcumin compared to diets containing only one of the compounds had a synergistic effect on reducing tissue damage and strengthening kidney function. No comparison, unfortunately, was made between the therapeutic response of thymoquinone and curcumin (Al Fayi et al., 2020).

Daphnetin is a derivative of coumarin that can be extracted from *Daphne odora*. Daphnetin has diverse biological effects, including kinase inhibition and antiproliferative and antioxidative properties. Among the applications reported for daphnetin are depletion of SLE symptoms, reduction of cerebral ischemia/reperfusion injury, and inhibition of LPS‐induced inflammatory enzymes (Liu et al., 2016). This bioactive substance suppressed OS in kidney tissue by enhancing the function of the Nrf2 and HO‐1 genes, which in turn reduced the expression of the inflammatory mediators TNF‐α and IL‐1β. Declines in BUN, creatinine, and MDA and improvement in histopathological changes confirmed the effects of daphnetin on reducing cisplatin renal toxicity (Zhang, Qin, et al., 2018; Zhang, Gu, et al., 2018).

Dioscin is a steroid saponin isolated from *Dioscorea nipponica* (Cho et al., 2013) and *Dioscorea zingiberensis* (Li, Cai, et al., 2010; Li, Huang, et al., 2010). In addition to its antitumor (Hsieh et al., 2013), antithrombotic, antihyperlipidemic (Li, Cai, et al., 2010; Li, Huang, et al., 2010), antiviral, and antifungal activity (Cho et al., 2013), dioscin has been used following cisplatin treatment to help reduce kidney injury. Dioscin has been shown to have positive results in an animal model, in a normal rat kidney tubular epithelial *cell* line (*NRK*‐*52E*), and a human renal proximal tubular epithelial *cell* line (*HK*‐*2*). Depletion of microRNA‐34a levels following dioscin exposure was reported to have affected the sirtuin (Sirt) 1/NF‐κB signal pathway leading to inhibition of the release of inflammatory cytokines such as TNF‐α, IL‐1β, and IL‐6. Nrf2 increased the production of protective cellular enzymes such as the glutamate‐cysteine ligase catalytic subunit (GCLC), glutamate‐cysteine ligase modifier subunit (GCLM), HO‐1, and SOD plus malondialdehyde (MDA*)*. Ultimately, these effects reduced oxidative stress and apoptosis (Zhang et al., 2017).

Embelin (2,5‐dihydroxy‐3‐undecyl1,4‐benzoquinone) is a member of the dihydroxy‐1,4‐benzoquinones class of compounds found in *Embelia ribes*. The bitter fruit of this herb has been reported to have therapeutic benefits for inflammatory diseases, fever, cancer, and a wide range of gastrointestinal and neurological disorders (Chaudhari et al., 2013; Gupta et al., 2013; Ko et al., 2018). Recent studies have demonstrated the effectiveness of embelin against cisplatin‐induced kidney toxicity following activation of Nrf2 /HO‐1 in rats. Nrf2 stimulated the synthesis of a series of antioxidant proteins such as HO‐1, GSH, GR, GST, CAT, and SOD and by affecting these inflammatory pathways, regulating the production of inflammatory mediators such as NF‐κB, TNF‐α, and IL‐1β. These changes have been shown to reduce the level of tissue damage markers and to improve kidney function tests such as creatinine clearance and blood nitrogen levels (Qin et al., 2019).

Farrerol is the main bioactive substance in the leaves of *Rhododendron dauricum*. Evaluation of the protective effect of this substance on certain liver disorders was associated with interesting results (Wang et al., 2019). Farrerol facilitated the entry of Nrf2 and its effect on the ARE gene, which in turn induced production of detoxifying enzymes such as HO‐1, GSH, NQO‐1, and SOD, reduced activation of the NF‐κB and NLRP3 genes, and suppressed phosphorylation of the mitogen‐activated protein kinase (MAPK). Decreased MAPK phosphorylation prevented triggering of p53, caspase‐3, Bcl‐2, Bax, and apoptosis. Farrerol can significantly reduce the ROS increase caused by cisplatin thereby improving kidney function markers such as BUN, Scr, KIM‐1, and NGAL (Ma et al., 2019).

6‐Hydroxy‐1‐methylindole‐3‐acetonitrile (6‐HMA) is a phytochemical found in the ethanol extract of the roots of *Brassica rapa*. The extract is efficient in managing some of the harmful complications of diabetes mellitus (Jung et al., 2008) and obesity (An et al., 2010). Treatment with 6‐HMA improved cisplatin‐induced renal damage by upregulating the expression of Nrf2 and by facilitating its nuclear translocation. In fact, activation of Nrf2 and its entry into the cell nucleus had a positive effect on ARE gene transcription and translational products of this gene, such as SOD, CAT, GR, HO‐1, MAD, and GSH (Moon et al., 2013).

Isoorientin (3′,4′,5,7‐tetrahydroxy‐6‐C‐glucopyranosyl flavone) is found in several plants such as *Phyllostachs pubescens, Gentiana patrinia* and in the tubers of *Pueraria tuberosa*. Anti‐inflammatory and antioxidant activity has been suggested for it (Anilkumar et al., 2017). Isorientin lowered renal impairment biomarkers by inducing Nrf2, increasing detoxification molecules such as HO‐1 and NQO‐1, and activating the Sirt1/Sirt6 pathway. Isoorientin, by facilitating the expression of Sirt1 and Sirt6 and subsequently stimulating Nrf2, increased the transcription of the ARE gene and suppressed MAPKs, NF‐κB, and p53 (Fan et al., 2020).

Kaempferol is a member of the flavonoid family of compounds found in vegetables such as kale, beans, tea, spinach, and broccoli. Antimalignant effects have been reported for kaempferol (Yoshida et al., 2008). Pretreatment with a kaempferol‐containing supplement enhanced Nrf2‐ARE interaction to inhibit NF‐κB and the MAPK/ERK apoptosis cascades via reducing the release of inflammatory cytokines such as NO, IL‐12, TNF‐α, and IL‐1β and neutralizing ROS. Changes in these pathways downregulated TPS3, Bax/Bcl‐2, caspase‐3,9, and poly ADP‐ribose polymerase (PARP), so kaempferol was able to ameliorate renal injury caused by cisplatin (Wang et al., 2020).

Lycopene is a bright red carotenoid hydrocarbon found in red fruits and vegetables, such as tomatoes, red carrots, watermelons, grapefruits, and papayas, but not in strawberries or cherries. Although lycopene is chemically a carotene, it has no vitamin A activity. Foods that are not red may also contain lycopene, such as asparagus, guava, and parsley. Lycopene is a precursor of vitamin A and has antioxidant activity (Hedayati et al., 2019). Lycopene has been reported to attenuate various pathophysiological conditions (e.g., malignancy). It acts through different mechanisms including (a) regulation of the insulin‐like growth factor (IGF)‐1/insulin‐like growth factor binding protein (IGFBP)‐3 system (Giovannucci, 1999), (b) upregulation of the expression of cell cycle regulatory proteins, (c) development of the gap‐junctional intracellular communication, and (d) modification of the gap‐junctional gene connexin 43 (Zhang et al., 1992). Lycopene also has been reported to affect redox signaling, protect against oxidative DNA damage, and prevent the destructive effects of IL‐6, androgen, and 5‐lipoxygenase. A lycopene supplementation improved the antioxidant defense system and reduced cisplatin nephrotoxicity involving Nrf2/HO‐1. Reinforcing this signal increased CAT, GPx, and SOD production and decreased the activation of several inflammatory factors by inhibiting NF‐κB compared to the absence of lycopene (Sahin et al., 2010).

Mangiferin is a nonsteroidal polyhydroxy polyphenolic molecule found in the mango fruit. Mangiferin has been shown to be effective in improving hepatic dysfunction (Pal et al., 2013). Mangiferin acts by upregulating Nrf2 via stimulation of phosphatidylinositol 3‐kinase (PI3K) and preventing caspase activation. To be more precise, mangiferin, by evoking PI3K and inducing nuclear translocation of Nrf2, improves the antioxidant capacity of the cell by increasing the expression of HO‐1, CAT, SOD, GR, and GST and modulating inflammatory molecules such as TNF‐α, IL‐1β, IL‐6, and IL‐10. It also has a preventive role against oxidative stress damage on the Golgi apparatus and mitochondria by suppressing the apoptosis chain of Bax/Bcl‐2 (Sadhukhan et al., 2018).

Piceatannol, a stilbene hydroxylated analog of resveratrol, is extracted from *Cassia garretiana* and *Rheum undulatum*. Although piceatannol and resveratrol share many properties, there is not sufficient evidence to suggest it is better than resveratrol. This NP prevented the development of various cancers and cardiovascular diseases (Seyed et al., 2016). Piceatannol showed a strong and generalized protective activity against renal injury induced by cisplatin. It reduced ROS production by stimulating the expression of antioxidant enzyme genes such as HO‐1, SOD, GST, GCLM, and GCLC, thereby inhibiting the NF‐κB inflammatory cascade and attenuating lipid peroxidation in an experimental animal model (Wahdan et al., 2019).

S‐allyl cysteine is an organosulfur compound in fresh garlic (*Allium sativum*). This plant (S‐allyl cysteine) has been shown to amplify the function of the immune system and to poses antibacterial, antiviral, and antifungal activity (Iciek et al., 2009). The protective effect of a single dose of S‐allyl cysteine against cisplatin‐induced nephrotoxicity was suggested to be related to the expression of Nrf2, protein kinase C‐β2 (PKCβ2), gp91^phox^, and p47^phox^. S‐allyl cysteine also increased the penetration of active Nrf2 into the cell nucleus resulting in ARE gene transcription of CAT, GPx, GR, SOD, and GSH, which ultimately stabilized cells to the oxidative‐induced effects caused by cisplatin (Gõmez‐Sierra et al., 2014).

*Schisandra sphenanthera* is another medicinal herb that is rich in bioactive chemicals such as schisandrin A, B, C, schisandrol A, B, and schisantherin A, B. This herb has been used to treat both cancer and hepatitis (Panossian & Wikman, 2008). An ethanol extract of *Schisandra sphenanthera* (Wuzhi tablet) prevented cisplatin‐induced renal damage by facilitating Nrf2 and antioxidant enzyme expression such as HO‐1, glutamate–cysteine ligase (GCL), NQO‐1, SOD, and GSH in *HK‐2* cells and mice. These enzymes attenuated the lipid peroxidation caused by free radicals, and eventually, renal biomarkers such as BUN and GFR were modified (Jin et al., 2015).

Sinapic acid is a small naturally occurring hydroxycinnamic acid, and a member of the phenylpropanoid family of chemicals found in the Brassicaceae. It has been suggested that this NP has antimicrobial, anti‐inflammatory, antioxidant, antihypertensive, anticancer, and antianxiety properties (Nićiforović & Abramovič, 2014). By targeting the signal Nrf2/ARE, sinapic acid corrected the levels of cellular detoxifying enzymes and NF‐κB proteins resulting in the production of chemokines (i.e., TNF‐α and IL‐6), MPO, caspase‐3, and the downregulation of Bax proteins. Pretreatment with sinapic acid in cisplatin‐consuming rats significantly improved renal function biomarkers including serum creatinine, urea, uric acid, and LDH and restored much of the histological impairment such as neutrophil infiltration in the renal tubules (Ansari, 2017).

Tetramethylpyrazine, also known as ligustrazine, is a chemical found in fermented cocoa beans and *Ligusticum wallichii*. When purified, tetramethylpyrazine is a colorless solid. It is classified as an alkylpyrazine. Tetramethylpyrazine is a phosphodiesterase suppressor, calcium antagonist, and antiplatelet aggregation mediator (Sheu et al., 2000). Suppression of high mobility group protein box 1 (HMGB1)/Toll‐like receptor 4 (TLR4) and stimulation of the Nrf2/ PPAR‐ɣ signaling were observed with this medication. In response to the induction of TLR4, there was an increase in the NF‐κB level with the release of chemokines. Nrf2 and PPAR‐ɣ, separately, improved renal toxicity by inducing changes in the inflammatory gene expression (Michel & Menze, 2019).

Xanthohumol is found in the female inflorescences of *Humulus lupulus* (hops). It belongs to a class of compounds that contribute to the bitterness and flavor of hops. (Dorn et al., 2012). Xanthohumol significantly improved ROS‐mediated cisplatin nephrotoxicity that appeared following the induction of Nrf2‐mediated phase‐II detoxifying enzymes including GSH, HO‐1, and SOD. In addition, suppression of TLR4 expression and NF‐κB phosphorylation reduced the release of inflammatory cytokines such as TNF‐α, IL‐12, IL‐6, and COX‐II and ultimately increased cell survival (Li et al., 2018).

### 3.3 Colistin

Colistin (polymyxin E) is an antibiotic used as a last resort for multidrug‐resistant Gram‐negative infections including pneumonia. Resistance to colistin first appeared in 2017. Colistin has significant renal and neurologic toxicity. Renal complications are manifested as a decrease in urine output with elevated blood urea nitrogen and serum creatinine, proteinuria, hematuria, and acute tubular necrosis (Abdelraouf et al., 2012).

Lycopene inhibited oxidative damage induced by colistin by stimulating the Nrf2/ARE/HO‐1 signaling pathway and blocking p38, MAPK, NF‐κB, Bax/Bcl‐2, and the caspase‐3 cascade. Lycopene plays a key role in the detoxification of oxidative damage caused by colistin (Dai et al., 2015). It also is effective in the recovery of renal disorders caused by colistin in a manner similar to its action in cisplatin nephrotoxicity.

### 3.4 Cyclophosphamide

Cyclophosphamide (cytophosphane) is a synthetic antineoplastic drug chemically related to nitrogen mustard. It is administrated to treat lymphoma, multiple myeloma, leukemia, ovarian cancer, breast cancer, small‐cell lung cancer, neuroblastoma, and sarcoma and to suppress the immune system. However, its use is restricted due to acute inflammation of the urinary bladder (cystitis) and renal injury (Takehiko et al., 1992).

An aqueous leaf extract of *Olea europaea* (olive) contains a large number of phenolic compounds such as rutin, apigenin, oleuropein, triterpenes, chalcones, and luteolin (Meirinhos et al., 2005). Abd El‐Azim reported the effective use of this plant in the treatment of hepatic toxicity caused by methotrexate (Abd El‐Azim, 2014). In a rat model of cyclophosphamide‐induced oxidative stress, an olive extract reduced the renal damage by activation of the Nrf2/HO‐1 pathway and reduction of inflammation and apoptosis. Increasing the amount of NQO‐1, SOD, CAT, and GSH along with decreasing IL‐1β, TNF‐α, Bax/Bcl‐2 (B‐cell lymphoma 2), and the caspase‐3 molecular cascade was suggested as involved mechanisms (Alhaithloul et al., 2019).

### 3.5 Cyclosporine

Cyclosporine A is a cyclic polypeptide immunosuppressant isolated from the fungus Beauveria *nivea*. It is used in postallogeneic organ transplants to reduce the activity of the patient's immune system and therefore the risk of organ rejection. Cyclosporine A also causes reversible inhibition of immunocompetent lymphocytes in the G0‐ and G1‐phase of the cell cycle. It is an antifungal, antirheumatic, immunosuppressive, and EC 3.1.3.16 (phosphoprotein phosphatase) inhibitor agent. However, its clinical use is limited due to its nephrotoxic properties (Grinyo & Cruzado, 2004).

Oleanolic acid is a pentacyclic triterpene carboxylic acid isolated from *Olea europaea* (olive). Pharmacological properties such as liver protection and anticancer and anti‐inflammatory properties have been reported for this substance (Liu, 1995). Oleanolic acid showed a generalized renoprotective effect against chronic cyclosporine nephropathy through metallothionein and the upregulation of the Nrf2/HO‐1 pathway. Activation of the Nrf2/HO‐1 pathway increased the levels of HO‐1, NQO‐1, SOD, GSH, S‐transferase, and GCL via influencing the ARE gene, thereby reducing degradation and apoptosis (Yaxin et al., 2014). Research in a mouse model has supported the possibility that oleanolic acid may be a potential therapeutic agent for the treatment of cyclosporine A‐induced nephrotoxicity involving Nrf2/ARE/HO‐1.

*Schisandra chinensis*, a member of the Magnoliaceae family, contains several potential therapeutic compounds including deoxyschizandrin, schizandrin, schizantherin A, and schizanhenol (Wang et al., 2013). Its therapeutic applications include control of renal failure, menstrual disorders, and neurological diseases, and chemical protection of the liver and heart (Chun et al., 2014). The complex of active ingredients in *S. chinensis* also has been reported to act as free radical scavengers, which increased mRNA and several detoxifying enzymes by activation of Nrf2 and regulating NF‐κB and p65. The *S. chinensis* extract has the potential to protect tubulointerstitial fibrosis, cell death, and the disruption of cyclosporine nephropathy. Expression of Nrf2/HO‐1/P‐glycoprotein genes including CAT, GPx, HO‐1, and autophagy‐related protein LC3A/B upregulation increased the antioxidant activity and cell life (Lai et al., 2017; Lai et al., 2015; Wu et al., 2018).

### 3.6 Epirubicin

Epirubicin is an anthracycline drug used in combination with other medications to treat breast cancer. Similar to other anthracyclines, epirubicin acts by intercalating DNA strands, resulting in a complex formation that inhibits DNA and RNA synthesis (Khasraw et al., 2012). Unfortunately, the renal toxicity of epirubicin limits its usefulness.

Paeonol (2′‐Hydroxy‐4′‐methoxyacetophenone) is a phenolic compound found in the herb *Moutan cortex*. It has been shown to have a variety of effects, such as treating liver cancer and heart damage (Chen et al., 2012; Li et al., 2012). Paeonol exerts its nephroprotective effects through upregulating the Nrf2/HO‐1 signaling pathway and facilitating HO‐1, GSH, GR, GST, CAT, and SOD biosynthesis. By impeding IKK/IκB phosphorylation and p65 nuclear translocation, paeonol reduced the transcription of the NF‐κB pathway genes that induced tissue inflammation and suppressed caspase‐9, caspase‐3, and Bax/Bcl‐2 in an animal model (Wu et al., 2017).

### 3.7 Gentamicin

Gentamicin is an aminoglycoside antibiotic used to treat Gram‐negative bacterial infections including bone infections, endocarditis, pelvic inflammatory disease, meningitis, pneumonia, urinary tract infections, and sepsis. Gentamicin, however, can cause ototoxicity and kidney problems following prolonged use. If used during pregnancy, it can harm the developing fetus (Ali et al., 2011).

*Actinidia deliciosa* (kiwi fruit) is a fruit rich in vitamins C, E, and K, potassium, magnesium, fiber, polyphenols, carotenoids, and flavonoids. The high concentration of these antioxidants plays an important role in maintaining a healthy lifestyle (Park et al., 2012), Liang and his colleagues have reported the antiproliferative effects of kiwi fruit on cancer cells (Liang et al., 2007). In vitro and in vitro evaluation showed that kiwi fruit restored renal function after gentamicin‐provoked necrosis and suppressed exponential growth of cancer cells. These findings suggested that the antiproliferative effects of kiwi fruit are most likely related to the Nrf2‐mediated antioxidant mechanism and the expression of NF‐κB and pro‐inflammatory proteins such as inducible nitric oxide synthase (iNOS), COX‐II, TNF‐α, and IL‐6 (Mahmoud, 2017).

Pinocembrin (5,7‐dihydroxyflavonone), a flavonoid, is an antioxidant found in damiana, honey, fingerroot, propolis, and the rhizomes of *Boesenbergia pandurata* (Punvittayagul et al., 2011). Pinocembrin has the potential for the treatment of cerebral ischemia, intracerebral hemorrhage, neurodegenerative diseases, cardiovascular diseases, and atherosclerosis (Shi et al., 2011). The nephroprotective effect of pinocembrin against gentamicin‐induced fibrosis was studied in vivo. Results showed that a treatment program containing pinocembrin controlled the renal pathogenesis through modulating the Nrf2/HO‐1 and NQO‐1 signaling pathways with activation of the organic anion transporter 3 (OAT3) mechanism thus improving renal function in rodents (Promsan et al., 2015).

Riceberry bran is obtained from Thai rice and contains a vitamin E complex (tocopherols and tocotrienols), β‐carotene, γ‐oryzanol, and anthocyanins (cyanidin 3‐glucoside and peonidin‐3‐glucoside). The anticancer effect of this bran extract in a human cancer cell line was investigated by Leardkamolkarn, et al. (Leardkamolkarn et al., 2011). The inflammation and necrotic effects of gentamicin on the kidneys were reduced. Arjinajarn and colleagues have speculated that increasing the expression of antioxidant enzymes such as SOD, CAT, NQO‐1, and HO‐1 regulated the protective properties of protein kinase C (PKC)/Nrf2 and facilitated OAT3 expression and repair of tubular protein function (Arjinajarn et al., 2016).

### 3.8 Methotrexate

Methotrexate (amethopterin) is a chemotherapy agent and an immune system suppressant used to treat breast cancer, leukemia, lung cancer, lymphoma, gestational trophoblastic disease, osteosarcoma, and rheumatoid arthritis (Vena et al., 2018). Methotrexate acts by blocking folic acid usage.

Berberine is a quaternary ammonium salt found in *Berberis vulgaris* (barberry), *Berberis aristata* (tree turmeric), *Mahonia aquifolium* (Oregon grape), *Hydrastis canadensis* (goldenseal), *Xanthorhiza simplicissima* (yellowroot), *Phellodendron amurense* (Amur cork tree), *Coptis chinensis* (Chinese goldthread), *Tinospora cordifolia*, *Argemone mexicana* (prickly poppy), and *Eschscholzia californica* (Californian poppy) (Imenshahidi & Hosseinzadeh, 2016). Some of the benefits of berberine include reducing liver damage (Iranshahy et al., 2018) and neurological disorders (Tavakkoli et al., 2019). Experiments in rats and cell viability tests have shown that berberine improved the texture of the kidney and the concentration of several serum biomarkers. Modulation of the Keap1/Nrf2 and NF‐κB/P_38_MAPK signaling pathways and the Bax/Bcl‐2/caspase‐3 protein has been suggested as the potential mechanism. Nrf2/HO‐1 cascade triggered the production of antioxidant enzymes such as SOD, CAT, NQO‐1, and HO‐1, suppressed the expression of COX‐II, TNF‐α, NF‐κB, and IL‐1β and inhibited apoptosis. Furthermore, berberine consumption attenuated oxidative stress, toxicity, and hemorrhage in the kidney after methotrexate administration (Hassanein et al., 2019).

Chicoric acid, obtained from a variety of herbs including *Ocimum basilicum, Cichorium intybus, and Echinacea purpurea,* is a phenolic product of the phenylpropanoid class of compounds and is a derivative of both caffeic acid and tartaric acid (Lee & Scagel, 2009). Chicoric acid has been used is to treat lead poisoning (Mu et al., 2019). Chicoric acid has been reported to activate the Nrf2/ARE antioxidative defense system and to increase the mRNA expression of SOD, CAT, GPx, GST, GR, NQO‐1, and HO‐1. Activation of this system prevented the biosynthesis of chemokine like IL‐1β by the damping of NF‐κB, the nucleotide‐binding domain (leucine‐rich‐containing family), and the pyrin domain‐containing the 3 (NLRP3) inflammasome axis. These alterations lead to attenuation of oxidative damage in the kidneys due to methotrexate (Abd El‐Twab et al., 2019).

C*ommiphora molmol*, a member of the Burseraceae family, is used in the production of myrrh which has been suggested as the active chemical in Giardia's diet (Mahmoud et al., 2019). An oleo‐gum resin extract of *C. molmol* has both anti‐inflammatory and antioxidant activity following Nrf2‐dependent activation of HO‐1, SOD, and CAT, NF‐κB downregulation, and inhibition of GPx, Bax/Bcl‐2, and caspase‐3. Its administration along with methotrexate prevented or at least reduced the renal toxicity in rats induced by methotrexate (Mahmoud et al., 2018).

Enoxolone (18 b‐Glycyrrhetinic acid) is a pentacyclic triterpenoid aglycone derivative of glycyrrhizin that is isolated from *Glycyrrhiza glabra* (licorice). It also has antioxidant and anti‐inflammatory properties (Eisenbrand, 2006). Enoxolone has been reported to be nephroprotective against the untoward effects of methotrexate by upregulating the Nrf2/ARE signaling pathway and increasing antioxidant enzymes including SOD, CAT, HO‐1, GPx, and GST in rats (Abd El‐Twab et al., 2016).

Ferulic acid, a hydroxycinnamic acid, is a phenolic phytochemical in the plant cell wall that can covalently bond to molecules such as arabinoxylans. The antioxidant properties of ferulic acid have led to its use in the treatment of cardiovascular diseases, diabetes, neurodegenerative diseases, and cancer (Srinivasan et al., 2007). Laboratory data indicated that ferulic acid provided beneficial properties for treatment of methotrexate‐induced renal disorder and acute kidney injury through Nrf2/ARE/HO‐1 upregulation, control of the NF‐κB inflammasome axis, a downstream molecular complex of NLRP3/caspase‐1/ASC, and modulating PPARγ expression (Mahmoud et al., 2019).

Formononetin is an isoflavone isolated from *Trifolium pratense* and *Astragalus membranaceus* that has been used as an adjuvant drug for cancer treatment (Kim et al., 2019). Formononetin targeted the Nrf2/Keap1 and PI3K/Akt signaling pathways and consequently increased the level of HO‐1, CAT, SOD, GSH, and GPx antioxidants. Formononetin also inhibited inflammatory mediators such as COX‐II, TNF‐α, NF‐κB, IL‐1β, and iNOS and the activity of the Bax/Bcl‐2 and caspase‐3 cascades. Supplements containing formononetin have been suggested as being beneficial in preventing or reducing the destruction of the renal epithelium (Aladaileh et al., 2019).

Vincamine, another natural indole alkaloid with potential medical properties, is isolated from *Vinca minor*. It is a vascular stimulant (Yin & Sun, 2011). A methotrexate treated animal model demonstrated that vincamine modulated the signs of renal disease and the level of selected inflammatory cytokines. The proposed pathway for the effect of vincamine was on the Nrf2 signal, followed by an incremental change in the levels of SOD, CAT, GPx, GST, and GR enzymes with a devaluation in the release of COX‐II, TNF‐α, NF‐κB, IL‐1β, and iNOS (Shalaby et al., 2019).

## 4 NRF2 AND TOXICANT‐INDUCED NEPHROTOXICITY

### 4.1 Arsenic

Arsenic is a toxic heavy metal and ubiquitous industrial pollution. Exposure to high levels of arsenic increases the risk of damage to various organs including skin, liver, lung, kidneys, brain, and the cardiovascular system (Flora, 2011).

Allitridin (diallyl trisulfide/DATS), a member of the organosulfur family of compounds, is produced by the hydrolysis of allicin in garlic. Many health benefits of garlic are attributed to DATS and include anticancer effects, platelet aggregation, blood pressure reduction, decreases in cholesterol levels, and increases in levels of reactive oxygen species (Powolny & Singh, 2008). DATS selectively kills cancerous cells in the prostate and breast, leaving healthy cells unharmed. This effect is attributed to increased reactive oxygen species (ROS) within cancer cells, increased number of cells that arrest in the G2 phase of mitosis, and increased caspase‐3 activity. Allitridin facilitated the entry of Nrf2 into the nucleus by activating the PI3K/Akt cascade. Akt attaches to Keap1 and separates from Nrf2. The activated Nrf2 upregulated ARE gene expression, enhanced the production of protective enzymes such as HO‐1, glutamylcysteine synthetase (GCS), and SOD, and reduced the NF‐κB level, which ultimately promoted cell survival against oxidative damage in renal tissue (Miltonprabu et al., 2017).

Sulforaphane, a compound within the isothiocyanate group of organosulfur compounds, is found in cruciferous vegetables such as broccoli, Brussels sprouts, and cabbage. It is produced when myrosinase transforms glucoraphanin, a glucosinolate, into sulforaphane following damage to the plant. A recent clinical study has suggested that sulforaphane can be used to treat chronic inflammatory diseases such as rheumatoid arthritis, diabetes, and cardiovascular injury (Mazarakis et al., 2020). Sulforaphane increased the production of phase‐II protective enzymes such as HO‐1, GSH, SOD, CAT, GPx, and GST by stimulating the PI3K/Akt signal and then inducing Nrf2 to enter the cell nucleus and upregulating the expression of the ARE gene. In addition, sulforaphane has been shown to have a direct inhibitory effect on thiobarbituric acid reactive substances (TBARS), lipid hydroperoxides (LOOH), and protein carbonyl content (PCC), preventing apoptosis and improving renal damage caused by arsenic (Thangapandiyan et al., 2019).

Tannic acid has been reported to have several therapeutic applications including anti‐neuroinflammatory effects. Commercial tannic acid is extracted from the following plant parts: Tara pods (*Caesalpinia spinosa*), gallnuts from *Rhus semialata* or *Quercus infectoria* or Sicilian sumac leaves (*Rhus coriaria*) (Wu et al., 2019). In addition to enhancing the immune response to some pathogens, tannic acid by affecting the Nrf2 molecular pathway, facilitated the synthesis of beneficial enzymes such as GSH, total sulphydryl, GST, SOD, MAD, and glucose‐6‐phosphate dehydrogenase (G6PDH). It also reduced the release of inflammatory cytokines such as IL‐6, IL‐8, and TNF‐α, inhibited the NF‐κB signal, diminished the expression of Bax/Bcl‐2 proteins, prevented apoptosis, and relieved kidney damage caused by arsenic (Jin et al., 2020).

### 4.2 Atrazine

Atrazine is an herbicide of the triazine class used to prevent pre‐emergence broadleaf weeds. It is a major source of drinking water pollution. Exposure to atrazine is associated with teratogenic events and endocrine, brain, liver, and heart disorders. Because of its accumulation in the kidneys, renal damage is a common outcome of atrazine exposure (Zhang, Qin, et al., 2018; Zhang, Gu, et al., 2018).

Lycopene has the potential to trigger AMPK to phosphorylate Nrf2. By promoting HO‐1 and NQO‐1 genes downstream, Nrf2 increased the level of phase‐II enzymes such as SOD, CAT, and GSH‐Px and lowered the autophagy‐related genes (Beclin‐1 and ATGs) and proteins (p62/SQSTM and LC3). This beneficial property of lycopene has been shown to reduce renal toxicity caused by atrazine (Lin, Bayarsengee, et al., 2018; Lin, Xia, et al., 2018).

### 4.3 Cadmium

Cadmium is a heavy metal environmental pollutant (Järup & Åkesson, 2009). The kidney is the main organ affected by chronic exposure because cadmium is preferentially absorbed by receptor‐mediated endocytosis in the kidneys where it binds to metallothionein and accumulates. Degradation of the cadmium–metallothionein complex by lysosomal enzymes releases cadmium into the cytosol, resulting in the release of ROS and cellular damage and apoptosis in renal tissue (Panel, E. C, 2009).

Carnosic acid (salvin) is a benzenediol abietane diterpene found in rosemary (*Rosmarinus officinalis*) and common sage (*Salvia officinalis*). Carnosic acid is used in the food, health, and cosmetics industries for its antioxidant and antimicrobial properties (Birtić et al., 2015). Experimental results have shown that administration of carnosic acid was effective in reducing cadmium‐induced renal toxicity. Carnosic acid prevented defects to DNA and other macromolecules by facilitating Nrf2, increasing SOD, CAT, GSH, GPx, and GR enzymes, and modulating HO‐1, transforming growth factor β (TGF‐β), and small molecules against decapentaplegic (Smad)‐3,7 (Das et al., 2019). Activation of both Nrf2 and Smad prevented inflammation and fibrosis by increasing the overall cellular antioxidant capacity and reducing cellular collagen production.

The seeds of *Vitis vinifera* (Grape) are rich in proanthocyanidins, a flavonoid that is a member of the family of polyphenolic compounds. Bagchi et al claimed that the proanthocyanidins were effective in controlling dysfunction of organs such as the heart (Bagchi et al., 2003). A grape seed proanthocyanidins extract increased the stability of renal cells against cadmium toxicity. The study demonstrated that inducing membrane‐bound ATPase and interaction of Nrf2 with ARE stimulated the synthesis of HO‐1, GCS, SOD, glutathione disulfide (GSSG), CAT, GPx, GST, and G6PD, and reduced the levels of inflammatory mediators such as TNF‐α, NF‐κB, IL‐1β, IL‐6, NO, and p65 (Nazima et al., 2015).

Resveratrol (3,5,4′‐trihydroxy‐trans‐stilbene) is a phytoalexin produced in several plants in response to physical damage or microbial attack. Resveratrol had a salutary effect in regulating the pathophysiology of some cardiovascular disorders, by slowing down the innate rate of aging (Baur & Sinclair, 2006). Its consumption was effective in reducing renal toxicity caused by cadmium. Elevated cell survival was hypothesized to occur by regulating Nrf2 (Zhang et al., 2020).

### 4.4 7,12‐Dimethylbenz[a]anthracene

7,12‐Dimethylbenz[a]anthracene (DMBA), a polycyclic aromatic hydrocarbon, is a carcinogenic organic pollutant and an immunosuppressor that is generated from the incomplete combustion of fossil fuels (Miyata et al., 2001). Renal damage is one of the harmful side effects of DMBA poisoning.

Tangeretin is a polymethoxylated flavone isolated from tangerines and other citrus peels with anticancer and cardiovascular and anti‐inflammatory activity (Benavente‐Garcia & Castillo, 2008). In a rodent study, tangeretin significantly increased the levels of both enzymatic and nonenzymatic antioxidants and decreased the inflammatory cytokines, lipid peroxides, and DNA damage markers. These effects have been suggested to be related to the regulation of Nrf2/Keap1 and its downstream effectors. Tangeretin increased the expression of the ARE gene due to its effect on Nrf2, thereby enhancing the levels of GSH, GST, and SOD and reducing OS markers such as MDA, peroxidase (POD), MPO, and iNOS (Lakshmi & Subramanian, 2014).

### 4.5 Lead

Lead has numerous industrial uses but, unfortunately, it also is a nondegradable environmental pollutant. Lead poisoning has many complications for various organs, especially the brain, blood, and kidneys, which can present with clinical symptoms such as abdominal pain, vomiting, joint and back pain, decreased learning ability, and anemia (Gidlow, 2004).

Luteolin is a flavone found in the leaves and bark of the aromatic flowering plant, *Salvia tomentosa*, clover blossoms, and ragweed pollen. Luteolin also occurs in the seeds of *Aiphanes aculeate*. Dietary sources of luteolin include celery, broccoli, green pepper, parsley, thyme, dandelion, perilla, chamomile tea, carrots, olive oil, peppermint, rosemary, navel oranges, and oregano. This NP has been used to treat obesity and insulin resistance (Xu et al., 2014). Pretreatment with luteolin triggered the binding of Keap1 and Nrf2, with the entry of this complex into the nucleus. The complex interacts with ARE resulting in the activation of HO‐1. Under these conditions, transcription of genes encoding for ROS scavenger compounds was facilitated and cellar protection was enhanced by increased production of SOD, CAT, GPX, and GR. The suppression of NF‐κB and the inactivation of MAPKs in the presence of luteolin downregulated the expression of TNF‐α and IL‐1β and enzymes (COX‐II and iNOS) involved in tissue inflammation. Luteolin reduced the level of apoptotic markers and alleviated the severity of the lead‐induced renal toxicity in rodents (Albarakati et al., 2020).

### 4.6 Mercury

Mercury is a heavy metal with industrial and agricultural applications. The elemental and organic forms of mercury exist as environmental pollutants and target mostly the CNS, liver, and kidneys (Bernhoft, 2012). One mechanism suggested for renal failure resulting from mercury is the suppression of Sirt1/PGC‐1α signaling. Mercury inhibited the Sirt1 deacetylation function, shutting down the PGC‐1α and Nrf2 axes and exacerbating oxidative stress. The result was an abnormality in the mitochondria associated with increased dynamin‐related protein 1 and decreased mitofusin 2. The mitochondrial dysfunction predisposed the renal cells to apoptosis (Li et al., 2019).

A *Ziziphus spina‐christi* extract contains beneficial compounds such as flavonoids, alkaloids, tannins, triterpenoids, phytosterols, saponins, and essential oils. The fruit and leaves were used in Ancient Egyptian both as food and medicine. The compounds extracted from the plant continue to be prescribed to decrease inflammation and to modulate metabolic syndrome and immune system disorders as well as for its antitumor and antiprotozoa properties (Dkhil et al., 2018). The proposed mechanism of the protective effect of this extract on mercury‐damaged kidneys is by elevating the expression of the Nrf2 gene resulting in the inhibition of Bax/Bcl‐2 and apoptosis. The key factor appears to be stimulation of phase‐II proteins such as CAT, GSH, GPX, GR, NQO‐1, and HO‐1, and the reduction of ROS and inflammatory modulators such as TNF‐α, IL‐1β, and NOS. Decreased DNA damage by cleavage of the DNA repair enzyme PARP and caspase‐3 induction may also be involved (Almeer et al., 2019).

### 4.7 Paraquat

Paraquat (N,N′‐dimethyl‐4,4′‐bipyridinium dichloride) is also known as viologen, a family of redox‐active heterocyclic compounds of similar structure. This herbicide is toxic to humans and animals due to its redox activity, which produces superoxide anions (Bacigalupo et al., 2005). Paraquat has been linked to the development of pulmonary fibrosis, Parkinson's disease, and renal and hepatic failure through involving mitochondria and excessive production of ROS (Ossowska et al., 2006; Tanner et al., 2011).

Cycloartenyl ferulate (CAF) is a triterpene alcohol extracted from rice bran oil‐derived γ‐oryzanol. γ‐Oryzanol containing CAF has been used in Japan for menopausal symptoms, mild anxiety, stomach upset, and high cholesterol. CAF has several unique biological activities such as blood‐cholesterol lowering, anticarcinogenicity, anti‐inflammatory, and antioxidant properties (Nagasaka et al., 2007). Data from a study on *HK‐2* cells suggested that CAF had a renal protective effect via stimulating Nrf2. The level of detoxification enzymes, inhibition of lactate dehydrogenase (LDH), cell death‐inducing enzymes (caspase), and cleavage of PARP, and improving mitochondrial function have been proposed as modes of action for CAF (Hong et al., 2013).

### 4.8 Sodium oxalate

Sodium oxalate can give rise to mesenchymal–epithelial transition disorder (EMT), a condition where epithelial cells lose polarity and cell–cell adhesion. In the case of both renal fibrogenesis and end‐stage renal disease (ESRD), EMT appears to be involved in the pathophysiology of the disease (Zeisberg & Kalluri, 2004).

Epigallocatechin gallate (epigallocatechin‐3‐gallate, EGCG) is the ester of epigallocatechin and gallic acid. The most abundant source is green tea (*Camellia synesis)*. Multiple applications have been proposed for EGCG, one of which is the prevention of oxalate‐induced EMT in Madin–Darby canine kidney (*MDCK*) cells via activation of the Nrf2 pathway. The involvement of Nrf2 activation is based on the following: (a) enhancing translation of antioxidants, for example catalase, (b) lowering immunoreactive proteins such as vimentin, and (c) increasing the level of zonula occludens‐1 (ZO‐1) or tight junction protein‐1 (Kanlaya et al., 2016).

### 4.9 Tert‐butyl hydroperoxide (t‐BHP)

Tert‐butyl hydroperoxide (t‐BHP) is an organic peroxidase and environmental pollutant that has renal toxicity. Rashid and his colleagues as well as other researchers continue to look for NPs that show a potential to modify renal function following t‐BHP poisoning (Rashid et al., 2013).

Mangiferin has been reported to protect kidney epithelial cells against t‐BHP damage by affecting Nrf2. Activation of this molecular cascade accelerated the production of protective mediators against cellular stress such as HO‐1, NQO‐1, CAT, GPx, GR, and GST. These events decreased the expression of inflammatory intermediaries and ROS in macrophages exposed to t‐BHP by suppressing the MAPK/NF‐κB signaling pathway (Saha et al., 2016).

Rutin (quercetin‐3‐O‐rutinoside, vitamin P) is a flavonoid extracted from citrus fruit. It is added to multivitamins and other drugs to control signs of blood and vascular disorders, hyperglycemia, and dyslipidemia (Fuentes et al., 2013). Rutin reduced the effects of t‐BHP on the kidneys in an in vitro study. It stimulated Nrf2, upregulated ARE gene expression, and increased antioxidant enzymes such as GPx, GST, GSH, SOD, and GR. In this study, rutin enhanced the antioxidant defense system against destructive cellular stresses such as ROS, NO, and iNOS (Singh et al., 2019).

### 4.10 Titanium dioxide (TiO_2_)

Titanium dioxide is the naturally occurring oxide of titanium, which is sourced from anatase, ilmenite, and rutile. TiO_2_ in the form of nanoparticles is used in food, cosmetics, and plastics. It has potential detrimental health effects because it can accumulate in the kidneys causing cellular damage and necrosis. TiO2 causes oxidative stress both by direct chemical reaction and by stimulating the onset of the inflammatory process that eventually leads to damage of cell membranes and other organelles such as mitochondria and damage to the DNA (Baranowska‐Wójcik et al., 2020).

*Moringa oleifera* (drumstick tree or horseradish tree) grows in many tropical regions. It contains significant amounts of protein, amino acids, vitamins A, B, C, and E, β‐carotene, minerals, and phenolic and flavonoid compounds. It has the potential to ameliorate leukemia and liver cancer (Khalafalla et al., 2010). Biochemical analyses and histopathological studies showed that a leaf extract reduced the effects of TiO_2_‐induced nephrotoxicity via upregulating Nrf2, HO‐1, and other detoxifying agents and then downregulating the expression of inflammatory factors such as KIM‐1, NF‐кB, TNF‐α, and heat shock protein 70 *(*Hsp70) (Abdou et al., 2019) (Table 1).

**TABLE 1:** Summary of useful NPs against nephrotoxicity of xenobiotics by activating Nrf2 pathway

The herbal(s) or microorganism(s)	Type of extract	Bioactive compounds/dose/route	Class	Molecular tests	Nephrotoxic agent and dose/route	Study model	Sources of NPs	Ref.
*Camelia sinensis*	Aqueous	15 or 45 mg kg^−1^ day^−1^ for 12 months/oral	Polyphenol	Nrf2; NQO‐1; HO‐1; GST‐α1; SOD1; SOD2; SOD3; CAT; GPx	Adriamycin 100 mg/kg/intraperitoneal	Sprague–Dawley rats	Herbal	(Espinosa et al., 2016)
*Polygonum multiflorum*	Ethanolic	2,3,5,40‐Tetrahydroxystilbene‐2‐O‐b‐D‐glucoside 2.5 and 10 mg kg^−1^ day^−1^ for 24 days/oral	Polyphenol	HO‐1; NQO‐1; Nrf2; FN; COL1A1; COL4A1; COL4A2; Podocin	Adriamycin 10 mg/kg/intravenous	Balb/c mice	Herbal	(Lin, Bayarsengee, et al., 2018)
*Nigella sativa*	Aqueous	Thymoquinone 50 mg kg^−1^ day^−1^ for 3 weeks/oral		Nrf2; GAPDH; MDA; SOD; GST; LPO; NOX4; TNF‐α; IL‐6; IL‐10	Adriamycin 3.5 mg/kg twice weekly/intraperitoneal	Sprague–Dawley rats	Herbal	(Elsherbiny & El‐Sherbiny, 2014)
*Andrographis paniculata*	Ethanolic	200 and 400 mg kg^−1^ day^−1^ for 7 days/oral		AOPP; H2O2; MDA; NO; MPO; NPT; PT; XO; TP urine; PC; SOD; GSH; GPx; GST	Cisplatin 10 mg kg^−1^ day^−1^ for 7 days/intraperitoneal	Wistar rats	Herbal	(Adeoye et al., 2018)
*Curcuma Longs Linn*		Curcumin 100 mg/kg/oral for 5 days	Polyphenol	TNF‐α; IL‐6; MRP‐1; KIM‐1; Akt; Nrf2; HO‐1; Caspase‐3; NFƙB; SOD; CAT; GR; GPx	Cisplatin 5 mg/kg/intraperitoneal	HEK 293 cell	Herbal	(Al Fayi et al., 2020)
*Daphne odora*		Daphnetin 2.5, 5, 10 mg/kg/for 3 days/intraperitoneal	Coumarin	MDA;IL‐1β; TNF‐α; Nrf2; HO‐1; p‐NF‐κB p65; NF‐κB p65; p‐IκBα; IκBα; ROS	Cisplatin 20 mg kg^−1^ day^−1^ for 4 days/intraperitoneal	C57BL/6 mice	Herbal	(L. Zhang, Gu, et al., 2018)
		Dioscin 10, 20 and 40 mg kg^−1^ day^−1^ for 12 days/oral	Steroid saponin	MDA; SOD; GSH; GSH‐Px; miR‐34a; Nrf2; HO‐1; GCLC; GCLM; AP‐1; COX‐II; IκB‐α; HMGB1; NF‐κB; IL‐1β; IL‐6; TNF‐α; Sirt1; Keap1	Cisplatin 7 mg/kg/intraperitoneal	Wistar rats and Normal rat kidney epithelial cell	Herbal	(Y. M. Zhang et al., 2017)
*Embelia ribes*		Embelin 25 and 50 mg kg^−1^ day^−1^ for 2 weeks/oral	Hydroxy benzoquinone	NF‐κB; IL‐1β; IL‐6; TNF‐α; Nrf2; HO‐1; GSH; GR; GST; CAT; SOD; MDA	Cisplatin 5 mg/kg/intraperitoneal	Sprague–Dawley rats	Herbal	(Qin et al., 2019)
*Rhododendron dauricum*		Farrerol 20 mg/kg/intraperitoneal		Cell viability; Nrf2; HO‐1; NQO‐1; Keap1; NOX4; p‐ JNK; T‐JNK; p‐ERK; T‐ERK; p‐p38; T‐p38; p‐ NF‐κB; p‐p65; NLRP3; p‐p53; p53; Bcl‐2; Bax; Caspase‐3	Cisplatin 20 mg/kg/intraperitoneal	C57BL/6 Mice and Mouse tubular epithelial cells (MTECs) and human proximal tubule cells (HK‐2)	Herbal	(Ma et al., 2019)
*Brassica rapa*	Methanolic	6‐Hydroxy‐1‐methylindole‐3‐acetonitrile 25, 50, or 100 μM for 3 hr/oral		Creatinine; LDH; Nrf2; HO‐1; SOD; CAT; GR; H2O2; MDA; GSH	Cisplatin 50 mM For 6 hr/intraperitoneal	Renal epithelial cell line derived from the porcine kidney	Herbal	(Moon et al., 2013)
*Phyllostachys pubescens, Gentiana, Patrinia and buckwheat*		Isoorientin 50 mg/kg for 3 days/intraperitoneal	Flavone	Cell viability; Sirt1; Sirt6; Nrf2; HO‐1; NQO‐1; Keap1; NOX4; p‐ JNK; T‐JNK; p‐ERK; T‐ERK; p‐p38; T‐p38; p‐NF‐κB; p‐p65; NLRP3; p‐p53; p53; Bcl‐2; Bax; Caspase‐3; MPO; MDA; SOD; GSH	Cisplatin 20 mg/kg/intraperitoneal	C57BL/6 Mice and Mouse renal tubular epithelial cells (mTECs)	Herbal	(Fan et al., 2020)
		Kaempferol 50, 100, 150 and 200 mg/kg for 2 weeks/oral	Flavonoid	Nrf2; HO‐1; TBARS; iNOS; GSH; SOD; CAT; GR; GST; NQO‐1; Caspase‐3; Caspase‐9; Bax; Bcl‐2; TP53; PARP; IL‐12; TNF‐α; MPO p38; Phosphorylated‐JNK; JNK phosphorylated‐p38; p38 Phosphorylated‐ERK1/2	Cisplatin 20 mg/kg/intraperitoneal	Balb/C mice	Herbal	(Wang et al., 2020)
*Solanum lycopersicum*		Lycopene 6 mg kg^−1^ day^−1^ for 10 days/oral	Carotenoid	Nrf2; HO‐1; β‐Actin; CAT; GPx; SOD; NF‐κB	Cisplatin 7 mg kg^−1^ day^−1^ for 10 day/intraperitoneal	Wistar rats	Herbal	(Sahin et al., 2010)
*Mangifera indica*		Mangiferin 5–30 µM/oral	Flavonoid	NDH; SDH; IL‐1β; TNFα; IL‐6; IL‐10; NF‐κB; Bax; Bcl‐2; Caspase‐3; Calpain; Caspase‐12; Nrf2; HO‐1; SOD‐2	Cisplatin 2, 5, 10, 15, 20, 25, 30, 40, and 50 µM in a serum‐free medium/oral	Swiss mice and Normal kidney epithelial (NKE) cell	Herbal	(Sadhukhan et al., 2018)
*Cassia garretiana and Rheum undulatum*		Piceatannol 10 mg kg^−1^ day^−1^ for 7 days/intraperitoneal	Hydroxylated analog of resveratrol	GCLC; GCLM; GST; HO‐1; KIM‐1; NGAL; Nrf2; NF‐κB; OCT‐2	Cisplatin 7 mg/kg/intraperitoneal	Wistar rats	Herbal	(Wahdan et al., 2019)
*Allium sativum*		S‐allylcysteine 6.25, 12.5, 15, 20, 25, 50, 100, 150 and 200 mg/kg On day 1 and 2, two doses On days 3 and 4, a single dose/intraperitoneal		MDA; Oxidized proteins; GSH; Nrf2; PKCβ2; p47^phox^; gp91^phox^; CAT; GPx; GR	Cisplatin 7.5 mg/kg/intraperitoneal	Wistar rats	Herbal	(Gõmez‐Sierra et al., 2014)
*Schisandra sphenanthera*	Ethanolic	Schisandrin 500 mg kg^−1^ day^−1^ Wuzhi tablet which contains 7.5 mg Schisantherin A per tablet for 10 days/intraperitoneal		NQO‐1; HO‐1; GCLC; GCLC; GSH; MDA; Nrf2; SOD	Cisplatin 15 mg/kg/intraperitoneal	NIH mice	Herbal	(J. Jin et al., 2015)
*Ligusticum wallichii*		Tetramethylpyrazine 50 and 100 mg kg^−1^ day^−1^ for 2 weeks/intraperitoneal		Bax; Bcl‐2; HMGB‐1; PPAR‐γ; KIM‐1; TLR4; Caspase‐3; Nrf2; HO‐1; NQO‐1; TNF‐α; IL‐1β; ATP	Cisplatin 7 mg/kg/intraperitoneal	Sprague–Dawley rats	Herbal	(Michel & Menze, 2019)
		Sinapic acid 20 mg/kg/for 10 days/oral		SOD level; Catalase activity; GSH; Nitric Oxide; lipid peroxidation MDA; total protein; TNF‐α; IL‐6; MPO; NF‐kB [p65]; Caspase; Bax 3; Bcl2; NRF2; HO‐1	Cisplatin 7 mg/kg/intraperitoneal	Wistar rats	Herbal	(Ansari, 2017)
*Humulus lupulus*		Xanthohumol 12.5, 25, 50 mg kg^−1^ day^−1^ for 3 days/intraperitoneal	Flavonoid	Nrf2; HO‐1; MDA; SOD; GSH; DCFH‐DA; DCF; TNF‐α; IL‐1ß; IL‐6; TLR4; NF‐κB; IκBα	Cisplatin 20 mg/kg/intraperitoneal	C57BL/6 mice	Herbal	(Li et al., 2018)
		Lycopene 5 and 20 mg kg^−1^ day^−1^ for 7 days/oral	Carotenoid	MDA; NO; iNOS; CAT; GSH; SOD; Caspase‐3; Caspase‐9; HO‐1; Nrf2; NF‐κB	Colistin 15 mg kg^−1^ day^−1^ for 7 day/intravenous	Mice	Herbal	(Dai et al., 2015)
*Olea europaea leaf*	Ethanolic	100, 150 and 200 mg kg^−1^ day^−1^ for 15 days/oral	Phenol	Bax; Bcl‐2; Caspase‐3; NF‐κB; NRF2; HMOX1; NQO‐1; Actb; MDA; Protein carbonyl; NO; GSH; SOD; CAT; GPx; TNF‐α; IL‐1ß	Cyclophosphamide 150 mg/kg/intraperitoneal	Wistar rats	Herbal	(Alhaithloul et al., 2019)
		Oleanolic acid 25 mg kg^−1^ day^−1^ for 7 days/intraperitoneal	Pentacyclic triterpenoid	Caspase‐3; α‐SMA; Nrf2; Keap1; HO‐1; NQO‐1; Bax; Bcl‐2; β‐Actin; SOD1; SOD2; CAT; MDA; 8‐iso‐PGF2α8‐OHdG	Cyclosporine 30 mg kg^−1^ day^−1^ For 4 weeks/subcutaneous injection	ICR mice	Herbal	(Hong et al., 2013)
*Schisandra chinensis*	Ethanolic	54,108 and 216 mg/kg/oral		MDA; CAT; GSH‐Px; Creatinine; BUN; P‐gp; Nrf2; HO‐1; GAPDH	Cyclosporine 25 mg/kg/oral	Sprague–Dawley rats	Herbal	(Lai et al., 2015)
*Moutan Cortex*		Paeonol 30 mg kg^−1^ day^−1^ for 4 days/intragastrical		SCR; GSH; GR; GST; CAT; SOD; NO; iNOS; TNF‐α; IL‐6; Cyt c oxidase; Caspase‐9; Caspase‐3; Bax; Bcl‐2; Nrf2; HO‐1; p‐NF‐κB; p‐IKKα/β; IκBα	Epirubicin 15 mg/kg/intravenous injection	Balb/c mice	Herbal	(Wu et al., 2017)
*Actinidia deliciosa*		37 g/kg/ for 8 days/oral	Polyphenols	Nrf2; NF‐kB; Na+; K+	Gentamicin 100 mg kg^−1^ day^−1^ For 8 weeks/intraperitoneal	Albino mice	Herbal	(Y. I. Mahmoud, 2017)
*Boesenbergia pandurata*	Ethyl acetate	Pinocembrin 50 and 75 mg kg^−1^ day^−1^ for 10 days/intraperitoneal	Flavonoids	Bax; Bcl‐xl; Caspase‐3; Nrf2; HO‐1; NQO‐1; PKCα; NOX4; MDA; SOD; Oat3; [3H]ES uptake	Gentamicin 100 mg kg^−1^ day^−1^ For 10 days/intraperitoneal	Sprague–Dawley rats	Herbal	(Promsan et al., 2015)
*Oryza sativa*	Methanolic	250, 500 or 1,000 mg kg^−1^ day^−1^ for 15 days/oral	Anthocyanins, β‐carotene, γ‐oryzanol, and vitamin E complex	[3 H]ES uptake; Oat3; PKC α; Nrf2; Keap 1; NQO‐1; HO‐1	Gentamicin 100 mg kg^−1^ day^−1^ For 15 days/intraperitoneal	Sprague–Dawley rats	Herbal	(Arjinajarn et al., 2016)
		Berberine 50 mg/kg/day for 10 days/oral	Alkaloid	Nrf2; Keap1; P_38_; MAPK; NFκB; β‐actin; NF‐κB; TBPS; GSH; SOD; NO; Bax; Bcl‐xl; Caspase‐3; Caco‐2; HepG2; Mcf‐7	Methotrexate 20 mg/kg/intraperitoneal	Albino rats	Herbal	(Hassanein et al., 2019)
*Cichorium intybus, Ocimum basilicum and Echinacea purpurea*		Chicoric acid 25 and 50 mg kg^−1^ day^−1^ for 15 days/oral gavage	Dicaffeyltartaric acid	NRF2; NQO‐1; HO‐1; Bax; Bcl‐2; β‐actin; KIM‐1; ROS; MDA; NO; TNF‐α; GSH; SOD; CAT; GPx; NF‐κB; NF‐κB p65; Caspase‐3; NLRP3; Caspase‐1 p20; IL‐1β	Methotrexate 20 mg/kg/intraperitoneal	Wistar rats	Herbal	(Abd El‐Twab et al., 2019)
*Commiphora molmol*	Ethanolic	125 and 250 mg kg^−1^ day^−1^ for 15 days/oral		Nrf2; NQO‐1; HO‐1; SOD; CAT; GPx; Bax; Bcl‐2; GSH; GPx; NF‐κB; TNF‐α; IL‐1β	Methotrexate 20 mg/kg/intraperitoneal	Wistar rats	Herbal	(A. M. Mahmoud et al., 2018)
		Ferulic acid 25 and 50 mg kg^−1^ day^−1^ for 15 days/oral	Hydroxycinnamic acid	Nrf2; NQO‐1; HO‐1; PPARγ; Bax; Bcl‐2; ROS; MDA; NO; SOD; CAT; GPx; Caspase‐3; NLRP3; Caspase‐1 p20; IL‐1β; NF‐κB p65; Cytochrome c	Methotrexate 20 mg/kg/intraperitoneal	Wistar rats	Herbal	(A. M. Mahmoud, Hussein, et al., 2019)
*Glycyrrhiza glabra*		18β‐Glycyrrhetinic acid 50 and 100 mg kg^−1^ day^−1^ for 7 days/oral	Triterpene	Nrf2; HO‐1; NO; TNF‐α; GSH; SOD; GPx; CST; Bax; Bcl‐2; β‐actin; KIM‐1	Methotrexate 20 mg/kg/intraperitoneal	Wistar rats	Herbal	(Abd El‐Twab et al., 2016)
*Trifolium pratense*		Formononetin 10, 20, and 40 mg kg^−1^ day^−1^ for 10 days/oral	Isoflavone	NF‐κB; IL‐1β; IL‐6; TNF‐α; COX‐II; Nrf2; HO‐1; GSH; GR; GST; CAT; SOD; MDA; LPO; TBARS; 8‐Oxo‐dG; NO; ATP; Caspase‐3; Caspase‐9; Cell viability	Methotrexate 20 mg/kg/at day 7/intraperitoneal	Wistar rats and HepG2 cells	Herbal	(Aladaileh et al., 2019)
		Vincamine 10, 20 and 40 mg/kg/oral	Alkaloid	Nrf2; HO‐1; MDA; SOD; CAT; NF‐κB; IL‐1β; IL‐10; TNF‐α; MPO; COX‐II; Caspase‐3	Methotrexate 20 mg/kg/intraperitoneal	Sprague–Dawley rats	Herbal	(Shalaby et al., 2019)
*Allium sativum*		Diallyl trisulfide 20, 40 and 80 mg/kg/day for 28 days/oral	Polysulfide	RBC; WBC; TBARS; LOOH; PC; ROS; NO; SOD; CAT; GPx; GST; GR; G6PD; GSH; T‐SH; Vitamin C; Vitamin E; Total ATPaseNa+/K+ ATPase; Ca2+ ATPase; Mg2+ ATPase	Arsenic 5 mg kg^−1^ day^−1^ for 4 weeks/intragastrically	albino rats	Herbal	(Miltonprabu et al., 2017)
*Cruciferous vegetables*		Sulforaphane 80 mg kg^−1^ day^−1^ for 28 days/oral	Isothiocyanate	GSH; total sulphydryl; Vitamin C; Vitamin E; SOD; CAT; GPx; GST; GR	Arsenic 5 mg kg^−1^ day^−1^ for 28 day/oral	Wistar rats	Herbal	(Thangapandiyan et al., 2019)
		Tannic acid 20 and 40 mg kg^−1^ day^−1^ for 10 days/oral	Polyphenol	CREA; CAT; GSH‐Px; SOD; MDA; GSH; total sulphydryl; GST; GR; G6PDH; LOOH; PC; CD; IL‐6; IL‐8TNF‐α; Bcl‐2; Bcl‐xl; p53; Bax; NF‐κB p65; Nrf2; Keap1	Arsenic 5 mg kg^−1^ day^−1^/intraperitoneal	Rats	Herbal	(W. Jin et al., 2020)
		Lycopene 5 mg/kg/ for 21 days/oral	Carotenoid	T‐AOC; T‐SOD; MDA; GSH; GSH‐Px; GSH‐ST; H2O2; CAT; Nrf2; NQO‐1; HO‐1; ATGs; Beclin‐1; LC3II/LC3I ratio; AKT1; MAPK1 p62/SQSTM; AMPK; p‐AMPK protein;	Atrazine 50 mg/kg and 200 mg/kg/ for 21 days/oral	Mice	Herbal	(J. Lin, Xia, et al., 2018)
*Rosmarinus officinalis and Salvia officinalis*		Carnosic acid 10 mg kg^−1^ day^−1^/oral	Diterpene	Cell viability; GSH; SOD; CAT; GPx; GST; GR; ROS; NO; H2O2; NADPH oxidase; TBARS; P‐Smad3; TGF‐β1; Smad7; Nrf2; Keap1; Cullin3; Ubiquitin; HO‐1; Collagen IV; Bcl‐2; Caspase‐3; Caspase‐8; Caspase‐9; IL‐6; IL‐1β; TNF‐α	Cadmium 4 mg kg^−1^ day^−1^/oral	Normal Kidney Epithelial (NKE) Cells and Mice	Herbal	(Das et al., 2019)
*Vitis vinifera*	Aqueous	Proanthocyanidin 100 mg kg^−1^ day^−1^ for 4 weeks/oral	Flavonoid	GSH; total sulphydryl; Vitamin C; Vitamin E; SOD; CAT; GPx; GST; GR; G6PD; iNOS; Caspase‐3; Total ATPases; Na+/K+ ATPase; Ca2+ ATPase; Mg2+ ATPase; TNF‐α; NO; IL‐6; IL‐1β; NF‐κB p65; LOOH; PCC	Cadmium 5 mg kg^−1^ day^−1^ for 4 weeks/oral	Wistar rats	Herbal	(Nazima et al., 2015)
		Resveratrol 400 mg/kg for 90 days/oral	Polyphenolic	T‐SOD; Cu‐Zn SOD; GSH‐Px; GST; CAT; T‐AOC; H2O2; MDA; CYP450; Cyt‐b5; APND; ERND; AH; NCR; Nrf2; SOD2; Sirt1; PGC‐1α; TFAM; Sirt3; PRDX3; VDAC1; Cyt C	Cadmium 140 mg/kg/oral	Chicken	Herbal	(Q. Zhang et al., 2020)
		Tangeretin 50 mg kg^−1^ day^−1^/oral	Flavonoid	Nrf2; Keap1; NQO‐1; HO‐1; NF‐κB; TNF‐a; IL‐1β; IL‐6; NF‐κB/p65; GAPDH; AST; ALT; ALP; Lipid peroxides Hydroperoxides; Protein carbonyls; Vitamin C; Vitamin E; GSH	7,12‐Dimethylbenz[a] anthracene 25 mg/kg/inhalation	Wistar rats	Herbal	(Lakshmi & Subramanian, 2014)
		Luteolin 50 mg/kg/oral	Flavonoid	SOD2; CAT; GPX1; GSR; NFE2L2; HMOX1; TNF‐a; IL‐1β; IL‐6; NOS2; Caspase‐3; Bax; Bcl‐2; LPO	Lead acetate 20 mg/kg/intraperitoneal	Rats	Herbal	(Albarakati et al., 2020)
*Ziziphus spina‐christi*	Methanolic	300 mg/kg for 28 days/oral		SOD2; GSR; NFE2L2; HMOX1; TNF‐a; IL‐1β; IL‐6; NOS2; Casp3; Bax; Bcl‐2; LPO; MDA; NO; GSH; CAT; GPx; GST; GR; NQO‐1; KIM‐1	Mercury chloride 0.4 mg/kg/intraperitoneal	Wistar rats	Herbal	(Almeer et al., 2019)
*Rice bran oil*		Cycloartenyl ferulate	Triterpene alcohol	Nrf2; HO‐1; NQO‐1; ROS; Superoxide; Bax; Bcl‐2; Caspase activity; LDH; PARP; Cell viability	Paraquat	Proximal tubular cell line derived from normal human kidney	Herbal	(G. L. Hong et al., 2013)
*Camellia Sinensis*	Aqueous	Epigallocatechin‐3‐gallate (EGCG) 25 μM	Polyphenol	Cell viability; E‐cadherin; Occludin; ZO‐1; Cytokeratin; Vimentin; Fibronectin; Nrf2; CAT	Oxalate 0.5 mM For 24 hr	MDCK renal tubular cells	Herbal	(Kanlaya et al., 2016)
		Mangiferin 20 mM		Cell viability; Nrf2; LDH; FRAP; MDA; GSH; GSSG; CAT; SOD; GPx; GST; GR; Caspase‐8; tBid; Bax; Cytochrome C; Apaf‐1; Caspase‐9; Caspase‐3; PARP	Tert‐butyl hydroperoxide 50 mM t‐BHP for 18 hr	Normal human kidney epithelial cells	Herbal	(Saha et al., 2016)
		Rutin 16.3–1.63 µM for 1 hr	Flavonoid	MDA; −CO; GSH; −SH levels; ROS; CAT; SOD; GPx; GST; GR; Nrf2; i;NOS	Tert‐butyl hydroperoxide 10 µM For 1 hr	Erythrocytes	Herbal	(Singh et al., 2019)
*Moringa oleifera*	Ethanolic	400 mg kg^−1^ day^−1^ for 2 mounts	Phenol	Nrf2; KIM‐1; HO‐1; TNF‐α; NF‐кB; HSP70; SOD; GST; GSH; GPx; Total thiols; MDA	Titanium dioxide 500 mg/kg	Albino rats	Herbal	(Abdou et al., 2019)

## 5 CONCLUSION

Understanding how NPs interact with cellular signaling pathways and altering the expression of genes will lead to a better insight into the treatment and potential prevention of chemical‐induced renal toxicity. This paper reviews information regarding several bioactive molecules found in NPs that have been reported to mitigate or reduce the severity of xenobiotic‐induced renal toxicity by affecting Nrf2. These NPs appear to play a pivotal role in detoxifying and protecting renal tissue against xenobiotic oxidative damage through stimulation of the Nrf2/ARE signaling pathway. Because Nrf2 upregulation is accompanied by antioxidant and anti‐inflammatory effects, activation of this signaling pathway may be involved in the antioxidant activity of these NPs. Keap1 inhibition and the facilitation of Nrf2 entry into the nucleus also have been suggested as important mechanisms contributing to Nrf2 activation by these NPs (Figure 1).

## CONFLICT OF INTEREST

The authors declare no conflicts of interest.

## Data Availability

The data that support the findings of this study are available on request from the corresponding author. The data are not publicly available due to privacy or ethical restrictions.
